# Chelidonin

**Published:** 1915-05

**Authors:** 


**Chelidonin,** Paul J. Hanzlik, Cleveland Med. Jour., Feb. 1915, was regarded by Meyer in 1892, as very similar to morphine but leaving no irritation of the central nervous system. A Pharmacologic study shows that it abolishes spontaneous contractions in the excised oesophagus, fundus and pylorus of frog's stomach, intestine of cat and rabbit, pregnant uterus of guinea-pig. It counteracts the effects of pilocarpine, pituitarin, histamin and barium chlorid. It relaxes frog's peripheral blood vessels previously contracted by epinephrin but has little effect upon untreated vessels. It antagonises the action of histamin on bronchial muscles. Other experiments are given, indicating that, excepting that it is not mydriatic, chelidonin depresses smooth muscle quite generally and should prove useful in asthma, various colics, etc.

